# Acceptance-Based Emotion Regulation Reduces Subjective and Physiological Pain Responses

**DOI:** 10.3389/fpsyg.2020.01514

**Published:** 2020-06-30

**Authors:** Valentina Haspert, Matthias J. Wieser, Paul Pauli, Philipp Reicherts

**Affiliations:** ^1^Department of Biological Psychology, Clinical Psychology, and Psychotherapy, Institute of Psychology, University of Würzburg, Würzburg, Germany; ^2^Department of Psychology, Education and Child Studies, Erasmus School of Social and Behavioural Sciences, Erasmus University Rotterdam, Rotterdam, Netherlands; ^3^Center of Mental Health (ZEP), University Hospital of Würzburg, Würzburg, Germany; ^4^Department of Medical Psychology and Sociology, Medical Faculty, University of Augsburg, Augsburg, Germany

**Keywords:** pain regulation, emotion regulation, acceptance, cognitive strategies, acute pain, acceptance-based strategy, psychological modulation of pain, pain ratings

## Abstract

Acceptance-based regulation of pain, which focuses on the allowing of pain and pain related thoughts and emotions, was found to modulate pain. However, results so far are inconsistent regarding different pain modalities and indices. Moreover, studies so far often lack a suitable control condition, focus on behavioral pain measures rather than physiological correlates, and often use between-subject designs, which potentially impede the evaluation of the effectiveness of the strategies. Therefore, we investigated whether acceptance-based strategies can reduce subjective and physiological markers of acute pain in comparison to a control condition in a within-subject design. To this end, participants (*N* = 30) completed 24 trials comprising 10 s of heat pain stimulation. Each trial started with a cue instructing participants to welcome and experience pain (acceptance trials) or to react to the pain as it is without employing any regulation strategies (control trials). In addition to pain intensity and unpleasantness ratings, heart rate (HR) and skin conductance (SC) were recorded. Results showed significantly decreased pain intensity and unpleasantness ratings for acceptance compared to control trials. Additionally, HR was significantly lower during acceptance compared to control trials, whereas SC revealed no significant differences. These results demonstrate the effectiveness of acceptance-based strategies in reducing subjective and physiological pain responses relative to a control condition, even after short training. Therefore, the systematic investigation of acceptance in different pain modalities in healthy and chronic pain patients is warranted.

## Introduction

Pain is an unpleasant sensory and emotional experience (Merskey and Bogduk, 2016), sometimes even referred to as an emotion that involves a physical sensation (Price, 1999; Wieser and Pauli, 2016). Thus, it is not surprising that emotion regulation (ER) strategies (Gross, 1998), which address the modification of affective experiences, are also capable of modulating the perception of pain (Masedo and Esteve, 2007; Braams et al., 2012; Kohl et al., 2013; Hampton et al., 2015). Numerous studies on commonly used ER strategies such as reappraisal and distraction (Gross, 2002; John and Gross, 2004) already demonstrated effective reductions of negative emotions (McRae et al., 2010; Kanske et al., 2011; Webb et al., 2012; Schönfelder et al., 2014) and pain (Van Damme et al., 2008; Verhoeven et al., 2011; Lapate et al., 2012; Hampton et al., 2015). The ability to regulate emotions was shown to correlate with the successful regulation of heat pain stimuli (Lapate et al., 2012), suggesting a general regulation skill for both emotion and pain.

A special case of ER are *acceptance-based strategies*, which are defined as the embracing of emotions or situations without judging or avoiding them (Hayes et al., 1999; Hofmann and Asmundson, 2008; Braams et al., 2012). The concept of acceptance-based strategies derives from the Acceptance and Commitment Therapy (ACT), a “third wave” cognitive and behavioral treatment approach, which focuses on contextual and experiential changes (Hayes et al., 2006; Hayes and Hofmann, 2017). The general goal of ACT is to increase *psychological flexibility* – the ability to stay present in the moment and to change or persist value-based behavior (Hayes et al., 2006). *Acceptance* (Hayes et al., 1999; Hofmann et al., 2009; Braams et al., 2012; Kohl et al., 2013) involves the active and aware embrace of events and is one of six core ACT processes underlying psychological flexibility (Hayes et al., 2006). Two closely related ACT processes and widely used conceptualizations of acceptance-based strategies in emotion and pain regulation research are *mindfulness* (“being present” and “non-judgmental”) (Braams et al., 2012; Kohl et al., 2013) and *cognitive defusion* (“decrease in believability of or attachment to an event”) (Kohl et al., 2013).

Even though acceptance-based strategies do not aim at the reduction of emotions or pain, various studies showed that they can alter the pain experience and therefore can be considered a regulation strategy (Kohl et al., 2012). Furthermore, there is an ongoing debate (Hofmann and Asmundson, 2008; Liverant et al., 2008; Hofmann et al., 2009; Wolgast et al., 2011) about the classification of acceptance within *the process model of ER* by Gross (Gross, 1998), suggesting that acceptance includes both antecedent- and response-focused components (for additional information on the conceptualization of acceptance, see Supplementary Material).

Previous studies found that acceptance-based strategies modulate behavioral pain measures such as pain threshold (PT) and tolerance more profoundly than other ER strategies – designed along the *process model of ER* – such as suppression of pain-related responses (Masedo and Esteve, 2007; Braams et al., 2012), reappraisal of the pain stimulus (Kohl et al., 2013), and distraction from pain (McMullen et al., 2008; Jackson et al., 2012; Moore et al., 2015). Similarly, so called *control-based protocols*, which are conceptualized as the exact opposite of ACT (Keogh et al., 2005) by instructing participants to ignore the pain stimulation and stop thinking about it, were found to be less effective in pain tolerance tasks than acceptance-based protocols (Keogh et al., 2005). A meta-analysis by Kohl et al. (2012) suggests that acceptance-based strategies compared to other regulation strategies are especially successful in increasing pain tolerance, while findings involving subjective pain measures such as pain intensity are less clear: acceptance-based strategies led to either decreased pain intensity compared to suppression (Masedo and Esteve, 2007) and control-based protocols (Gutierrez et al., 2004; Keogh et al., 2005), showed no difference when compared to distraction (McMullen et al., 2008; Moore et al., 2015), reappraisal (Kohl et al., 2013), or control-based protocols (Hayes et al., 1999; Paez-Blarrina et al., 2008a, b), or were even less effective than distraction (Kohl et al., 2013).

Most importantly, previous studies often used pre-to-post measurements or control conditions containing either spontaneous coping (Masedo and Esteve, 2007; Evans et al., 2014; Forsyth and Hayes, 2014) or no instructions at all (McMullen et al., 2008; Paez-Blarrina et al., 2008a; Braams et al., 2012). This might have led to an unsystematic use of ill-defined strategies and thus compromised the results. Some studies (Gutierrez et al., 2004; Keogh et al., 2005; Paez-Blarrina et al., 2008a; Kohl et al., 2013) even used no control condition at all, which makes it difficult to determine the actual effectiveness of a regulation strategy. Therefore, we chose to develop and include a neutral control condition to ascertain the effectiveness of acceptance-based strategies.

Only one study so far (Braams et al., 2012) implemented physiological measures to capture the effectiveness of acceptance-based strategies in modulating autonomous pain responses but used a between-subject design. A within-subject design might be better suited to account for potential inter-individual variance regarding physiological responses and regulation skills, which we consequently applied in our study.

In the present study, we compared an acceptance-based strategy with a carefully introduced control condition, where participants should not use any strategies, in a within-subject design. We complemented subjective measures of pain (intensity, unpleasantness) with psychophysiological pain responses (heart rate, HR; skin conductance, SC) (Rhudy et al., 2009; Loggia et al., 2011).

Our main goal was to test the successful reduction of experimentally induced pain by acceptance-based regulation. Thus, we hypothesized the acceptance-based strategy to result in decreased pain ratings and pain-evoked HR and SC responses compared to the control condition.

## Methods

### Participants

An optimal sample size of 27 participants was calculated *a priori* using G^∗^Power (Faul et al., 2009) assuming a medium to large effect size of Cohen’s d of 0.5 (Braams et al., 2012), alpha error of 0.05 (one-tailed paired *t*-test) and power of 0.8 (Kohl et al., 2013). Potential drop-out was considered and 31 (17 women) participants were recruited via an online platform by the University of Würzburg. They received either course credit or €10 for participation. Participants did not take any central nervous or pain medication and had no current or prior history of chronic pain (self-report). One participant indicated close to no pain sensation (pain intensity: *M* = 0.67, pain unpleasantness: *M* = 0.33; VAS 0–100) throughout the entire experiment and was therefore excluded from the final analysis. Thus, 30 participants (16 women; age *M* = 25.37, *SD* = 3.58) remained in the statistical analysis. The experimental procedure was conducted in accordance with the Declaration of Helsinki and approved by the institutional review board of the medical faculty of the University of Würzburg. All subjects gave written informed consent before participating.

### Thermal Pain Stimulation

Pain stimuli were delivered via a thermal stimulator with an active thermode area of 25 × 50 mm (Somedic SenseLab AB, Sösdala, Sweden). The thermode was attached to the volar forearm of the non-dominant hand. We assessed the individual pain threshold (PT) using the method of adjustment (Horn-Hofmann and Lautenbacher, 2015) to take individual differences in pain sensitivity (Nielsen et al., 2009) into account. For that, we instructed participants to adjust the thermode’s temperature – starting at 35°C – by pressing two different buttons (± 0.5°C/keystroke; maximum temperature 49°C) until they reached a level of thermal sensation that went from hot to just painful. This procedure was repeated three times and the average of all three temperatures was used as the final PT (*M* = 44.87°C, *SD* = 2.06). During practice trials and the main experiment, pain stimuli were calibrated to the individual PT plus 1°C (target temperature) to achieve a moderate but painful stimulation (Lautenbacher et al., 1995; Horn et al., 2012; Reicherts et al., 2016). Heat stimulation started at a baseline temperature of 10°C below PT and rose at a rate of 5°C/s. Thus, the thermode reached the target temperature after 2.2 s. The target temperature was presented for 10 s. Afterward, the thermode cooled down in 2.2 s to the baseline temperature. The pain stimulus duration of 10 s was chosen following similar experimental designs (Lapate et al., 2012; Prins et al., 2014; Hampton et al., 2015) and was supposed to give the participants sufficient time to engage in the strategy. To prevent habituation to the pain stimulus, the position of the thermode was changed after the PT procedure, after the practice trials and after each 6th trial of the main experiment (starting position was counterbalanced across participants).

### Instructions

The acceptance-based strategy was conceptualized along three core ACT processes, namely acceptance, mindfulness and cognitive defusion. Participants were instructed that acceptance involves the allowing of any experiences (*acceptance*) (Hayes et al., 2006) without further evaluation (*mindfulness*) (Braams et al., 2012). When participants saw the word “ACCEPT” on the screen, they should let their feelings run their natural course, allow themselves to stay with their emotions (Hofmann et al., 2009) and might employ the “clouds in the sky”-metaphor (Kohl et al., 2013) as a method of detachment from pain (*defusion*) and to facilitate understanding of the strategy. In the control condition “PERCEIVE,” participants were instructed to sense the pain as it is and not use any strategies. To underscore the distinction between conditions, instructions were briefly summarized: whenever the word “ACCEPT” appeared on the screen, participants should apply the acceptance-based strategy, while no strategy should be used when the word “PERCEIVE” appeared.

### Measures

#### Pain Ratings

Participants were instructed about the distinction of pain intensity and pain unpleasantness using the radio metaphor by Price et al. (1983). During the experiment, participants rated the heat pain stimuli using a digitized visual analog scale (VAS) presented on the screen, ranging from 0 = no pain/not unpleasant at all to 100 = maximum pain/extremely unpleasant, respectively.

#### Heart Rate

To measure electrocardiography (ECG), three electrodes were attached on the torso of the participant (right collarbone, left lower costal arch, left lower side of the torso). The continuous raw ECG-signal was sampled with 250 Hz, using a V-Amp amplifier and Brain Vision Recorder, V-Amp Edition 1.10, recording software (both Brain Products Inc., Munich, Germany). The signal was filtered (High cut-off: 30 Hz, Notch filter: 50 Hz) (Boucsein, 2012), R-waves were automatically detected and manually checked, the inter-beat intervals were calculated and then converted into the continuous HR (Koers et al., 1999) by the Vision Analyzer software (BrainProducts, Munich, Germany). HR signal was baseline corrected relative to 1 s interval before visual cue onset.

The effectiveness of ER might underlie temporal characteristics such as different strategy onsets, but only few studies have considered temporal dynamics so far (Dan-Glauser and Gross, 2011, 2015; Pavlov et al., 2014; Koval et al., 2015). To capture these, 25 1-s time bins were calculated (Dan-Glauser and Gross, 2015; Koval et al., 2015) by averaging intervals of 1 s, starting at cue onset (second 0) and ending with the offset of the fixation cross (second 25). A broad time interval was analyzed to capture potentially delayed psychophysiological responding following heat pain administration (Loggia et al., 2011). One participant was excluded from psychophysiological analyses due to bad data quality.

#### Skin Conductance

SC was recorded using two 8 mm Ag/AgCl surface electrodes (electrode gel: 0.5% NaCl) attached to the thenar and hypothenar eminence of the participant’s non-dominant hand. Similar to the ECG signal, the SC signal was sampled with 250 Hz, with constant application of 0.5 V. The signal was filtered (High cut-off: 1 Hz, Notch filter: 50 Hz) (Boucsein, 2012) and baseline corrected relative to 1 s interval before visual cue onset via Vision Analyzer software (BrainProducts, Munich, Germany). Again, 25 1-s bins were calculated to capture potential variations across trial duration, equally to the HR analysis. One participant was excluded from psychophysiological analyses due to bad data quality.

#### Questionnaires

Participants completed several questionnaires addressing habitually preferred ER styles [AAQ-II (Bond et al., 2011; Hoyer and Gloster, 2013), ASQ (Hofmann and Kashdan, 2010; Graser et al., 2012), ERQ (Gross and John, 2003; Abler and Kessler, 2009)], negative affect [STAI (Laux et al., 1981; Spielberger et al., 1983)], attitudes toward pain [FPQ-III (McNeil and Rainwater, 1998; Baum et al., 2013), PCS (Sullivan et al., 1995; Meyer et al., 2008), PSQ (Ruscheweyh et al., 2009)], optimism [LOT-R (Scheier et al., 1994; Glaesmer et al., 2008)] and resilience [RS-11 (Wagnild and Young, 1993; Schumacher et al., 2005)], which are supposed to affect pain and emotion processing, respectively (Rhudy and Meagher, 2000; Rhudy et al., 2004; Forys and Dahlquist, 2007; Geers et al., 2010; Hanssen et al., 2013; Boselie et al., 2014; Hampton et al., 2015; Moore et al., 2015; Biggs et al., 2016; Wieser and Pauli, 2016; Goubert and Trompetter, 2017; Hemington et al., 2017; Hinkle and Quiton, 2019). Questionnaires on ER styles were filled out before the experiment. All remaining questionnaires were presented after the experiment. Mean questionnaire scores and standard deviations are shown in Table 1.

**TABLE 1:** Mean questionnaire scores (M) and standard deviations (SD).

Questionnaire	Scale	*N*	*M*	*SD*
AAQ-II	Total	30	16.37	7.21
ASQ	Concealing/Suppression	30	2.95	0.61
ASQ	Adjusting/Reappraisal	30	3.12	0.66
ASQ	Tolerating/Accepting	30	3.76	0.47
ERQ	Cognitive reappraisal	30	4.59	0.88
ERQ	Expressive suppression	30	3.33	1.01
FPQ-III	Total	30	76.93	16.19
LOT-R	Pessimism	30	4.00	2.32
LOT-R	Optimism	30	8.97	2.57
PCS	Total	30	14.87	7.25
PSQ	Total	29	3.67	1.24
RS-11	Total	30	59.00	8.15
STAI	State	30	38.57	8.15
STAI	Trait	30	37.70	8.79

**AAQ-II, Acceptance and Action Questionnaire II; ASQ, Affective Style Questionnaire; ERQ, Emotion Regulation Questionnaire; FPQ-III, Fear of Pain Questionnaire-III; PCS, Pain Catastrophizing Scale; PSQ, Pain Sensitivity Questionnaire; RS-11, Resilience Scale 11; STAI, State-Trait Anxiety Inventory; LOT-R, Life-Orientation-Test Revised.**

#### Regulation Ratings/Manipulation Check

After each acceptance trial, participants rated how well they were able to regulate pain by applying the strategy (VAS 0–100; 0 = not at all; 100 = very well). As participants should not regulate pain in the control condition, no regulation ratings were taken. After the experiment, participants filled out a manipulation check survey (MCS) asking on a 9-point rating scale how clear the instructions were (1 = unclear, 9 = clear), how easily they could be implemented (1 = not at all, 9 = very well) and whether participants tried to distract themselves from pain during the main experiment (1 = not at all, 9 = very much).

### Procedure

Participants were informed about the details of the experiment and signed a written informed consent. They filled out questionnaires (STAI-S, ERQ, ASQ, and FAH-II) and answered a sociodemographic survey. As soon as they completed the questionnaires, the individual PT was assessed. Afterward, the electrodes for ECG and SC measures were attached. Participants received written standardized instructions on a screen describing the two experimental conditions (“ACCEPT” vs. “PERCEIVE”) and practiced each of them twice. The experimenter made sure that participants fully understood the instructions before starting the main experiment. Participants were separated from the experimenter by a folding screen and interacted with the experimenter solely for the relocation of the thermode. Each trial started with a central fixation cross on a gray screen. After 5 s, either the word “ACCEPT” or “PERCEIVE” appeared in the middle of the screen (cue onset), indicating the two conditions, respectively. The cue remained on the screen for 20 s before disappearing (cue offset). Five seconds after cue onset, the pain stimulation started. After cue and pain offset, a fixation cross was presented for 5 s, followed by the pain intensity and unpleasantness ratings, and the regulation ratings (acceptance only). The subsequent interstimulus interval varied between 15 and 18 s (randomly). The experiment consisted of 24 randomized trials (12 per condition, no more than two trials of the same condition in a row). After the experiment, participants filled out the remaining questionnaires (FPQ-III, PSQ, PCS, STAI-T, LOT-R, RS-11) and the MCS. The experimental procedure was controlled using the software Presentation (Version 17.2, Neurobehavioral Systems Inc., Albany, CA, United States).

### Statistical Analysis

Pain ratings (intensity and unpleasantness) were analyzed separately with pairwise *t*-tests comparing the acceptance vs. control condition. Pain intensity and unpleasantness ratings were compared with each other using pairwise *t*-tests of z-standardized difference scores between control and acceptance condition. Cohen’s *d*_*av*_ was used as a measure of effect size (Cohen, 1988) as recommended by Lakens (2013). For analysis of HR and SC, we used a repeated-measures ANOVA with the within-factor condition (acceptance vs. control) and the within-factor time (twenty-five 1-s bins) and reported partial eta-squared η*_*p*_^2^*. In case the assumption of sphericity was violated (Mauchly), the Greenhouse-Geisser correction was applied. *Post hoc* comparisons of different factor levels were realized using pairwise *t*-tests. Pearson correlations were conducted to explore the association of pain ratings during the acceptance-based strategy and questionnaire scores (ERQ, ASQ, AAQ-II, STAI, PCS, FPQ-III, PSQ, LOT-R, and RS-11). The regulation ratings were analyzed using a repeated-measures ANOVA with the within-factor trials (4 levels) by averaging three successive trials. Significance level was defined as *p* < 0.05.

## Results

### Pain Ratings

**FIGURE 1:**
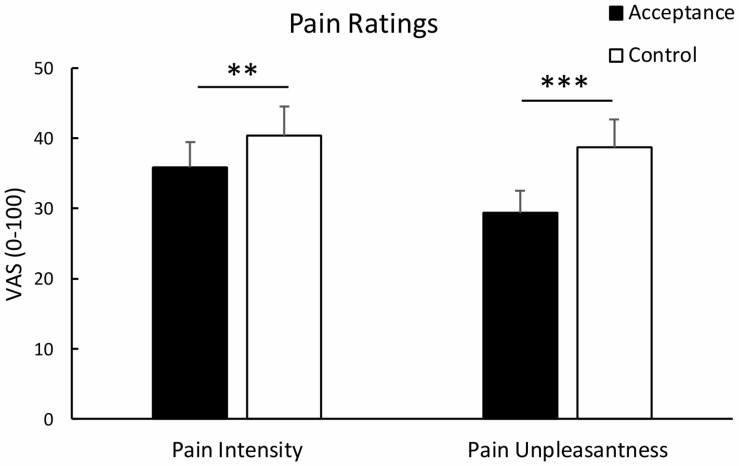
Mean pain intensity and unpleasantness ratings with standard error bars for the acceptance and control condition. Both pain intensity and pain unpleasantness were significantly lower in the acceptance than the control condition. **p < 0.01; ***p < 0.001.

Analysis of pain intensity revealed a significant effect of *condition*, *t*(29) = 3.23, *p* = 0.003, *d*_*av*_ = 0.217, indicating lower pain intensity ratings for the acceptance compared to the control condition. Similarly, analysis of pain unpleasantness revealed a significant effect of *condition*, *t*(29) = 5.26, *p* < 0.001, *d*_*av*_ = 0.484, indicating reduced pain unpleasantness ratings for the acceptance vs. control condition. Mean pain intensity and unpleasantness ratings are shown in Figure 1. Analysis of standardized difference scores yielded a stronger regulatory effect of acceptance for unpleasantness than for intensity pain ratings, *t*(29) = -3.09, *p* = 0.004, *d_*av*_* = -0.486.

### Heart Rate

**FIGURE 2:**
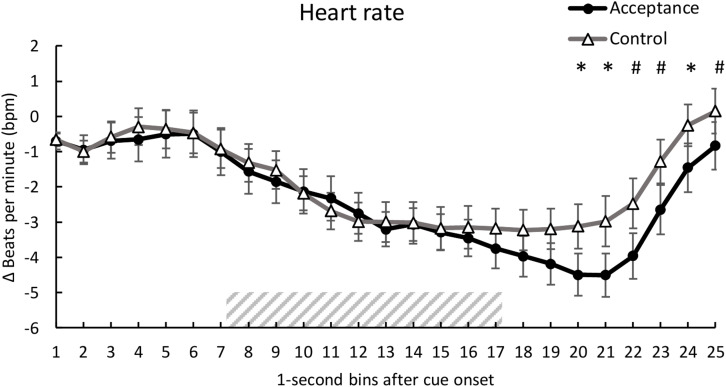
Mean time course (1-s bins) of the heart rate (baseline-corrected 1 s before cue onset) with standard error bars for the acceptance and control trials. The dashed area represents the 10-s heat pain stimulus (7.2 s until 17.2 s after cue onset). There was a significantly lower HR for acceptance compared to the control trials during seconds 20, 21, and 24 of the trial. *p < 0.05; ^#^*p* < 0.10.

Analysis of HR revealed no significant main effect of *condition*, *F*(1, 28) = 0.76, *p* = 0.390, η*_*p*_^2^* = 0.027, but a significant main effect of *time*, *F*(3.96, 110.98) = 17.14, *p* < 0.001, η*_*p*_^2^* = 0.380 and a significant interaction of *condition* and *time*, *F*(6.34, 177.56) = 2.46, *p* = 0.024, η*_*p*_^2^* = 0.081. *Post hoc* analyses revealed lower HR for the acceptance condition compared to the control condition [second 20, *t*(28) = -2.10, *p* = 0.045; second 21, *t*(28) = -2.22, *p* = 0.035; second 22, *t*(28) = -2.00, *p* = 0.056; second 23, *t*(28) = -2.03, *p* = 0.052; second 24, *t*(28) = -2.12, *p* = 0.043, 25; *t*(28) = -1.73, *p* = 0.094]. The mean time course for both conditions is shown in Figure 2.

### Skin Conductance

**FIGURE 3:**
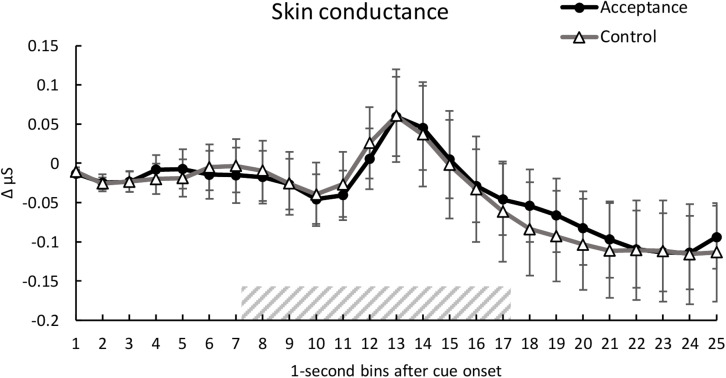
Mean time course (1-s bins) of the skin conductance (baseline-corrected 1 s before cue onset) with standard error bars for the acceptance and control trials. The dashed area represents the 10-s heat pain stimulus (7.2 s until 17.2 s after cue onset). There were no significant differences between the two conditions over time.

Analysis of SC showed no significant main effect of *condition*, *F*(1, 28) = 0.10, *p* = 0.920, η*_*p*_^2^* < 0.01. A significant main effect of *time* was found, *F*(1.94, 54.35) = 4.01, *p* < 0.001, η*_*p*_^2^* = 0.125, indicating a SC reaction to the heat pain stimulus (see Figure 3). There was no significant interaction between *condition* and *time*, *F*(2.97, 83.20) = 0.30, *p* = 0.846, η*_*p*_^2^* = 0.01.

### Correlations of Pain Ratings and ER Style Questionnaires

Correlation analysis revealed no significant associations between pain ratings of the acceptance condition and ER style questionnaire scores (ERQ subscales reappraisal and suppression; ASQ subscales suppression, reappraisal and accepting; AAQ-II total score) nor the resilience scale RS-11 total score; all *p*s > 0.063. There were also no significant correlations between the acceptance ratings and the remaining questionnaire scores (STAI state & trait, PCS total, FPQ-III total, PSQ total, LOT-R optimism & pessimism; all *p*s > 0.083).

### Regulation Ratings/Manipulation Check

Analysis of regulation ratings did not show a significant change over *time*, *F*(3, 87) = 2.48, *p* = 0.066, η*_*p*_^2^* = 0.079. However, there was a trend indicating better subjective regulatory performance toward the end of the experiment: Trials 1–3: *M* = 60.04, *SD* = 19.72; trials 4–6: *M* = 58.53, *SD* = 19.40; trials 7–9: *M* = 61.34, *SD* = 16.45; trials 10–12: *M* = 64.67, *SD* = 17.79.

In the MCS, participants rated the instructions of acceptance, *M* = 7.80, *SD* = 1.10, and the control condition, *M* = 8.33, *SD* = 0.84, as rather clear and easy to implement (acceptance: *M* = 6.87, *SD* = 1.38; control *M* = 7.97, *SD* = 1.19). Further, participants did not distract themselves from heat pain (*M* = 3.43, *SD* = 2.11).

## Discussion

In the present study, we found that an acceptance-based pain regulation strategy led to a reduced perception of acute heat pain compared to a carefully instructed control condition as indicated by pain intensity and unpleasantness ratings. Also, HR was significantly lower during acceptance-based regulation of pain, while SC responses showed no significant difference between conditions. The present study demonstrates that acceptance-based strategies can modulate subjective and physiological correlates of pain in healthy controls even after brief practice.

### Modulation of Pain Ratings by the Acceptance-Based Strategy

Acceptance compared to the control condition led to significantly reduced pain ratings, replicating previous findings (Gutierrez et al., 2004; Keogh et al., 2005; Masedo and Esteve, 2007; Paez-Blarrina et al., 2008a, b; Braams et al., 2012; Kohl et al., 2013). Especially pain unpleasantness was sensitive for the use of the acceptance-based strategy, as indicated by the significant difference across pain rating dimensions.

The pronounced modulation of the affective component of pain is in line with the theoretical foundation of acceptance-based strategies, which aim at changing the behavioral and emotional pain responses rather than its sensory experience (Hayes et al., 1999; Masedo and Esteve, 2007; Kohl et al., 2013). Nevertheless, we found that accepting the heat pain stimulation also decreased sensory aspects of pain. These results resemble the findings by Prins et al. (2014) who showed that a brief mindfulness induction (comprising acceptance-based strategies) led to stronger reductions of pain unpleasantness than pain intensity but only in high pain catastrophizers. The authors point out that the aim of mindfulness is not the reduction of symptoms but instead modifying the experience of the symptoms (Chiesa and Serretti, 2011; Prins et al., 2014), which is likely also the case in acceptance-based strategies.

Kohl et al. (2012) concluded in their meta-analysis that acceptance-based strategies probably are most effective at modulating behavioral pain measures whereas findings concerning pain ratings are rather inconsistent. This heterogeneity might be due to the combination of a pain tolerance task and the subsequent needless measure of pain ratings. One study (Kohl et al., 2013), for instance, found elevated pain tolerance markers *and* higher pain intensity ratings for a pain acceptance condition. Some previous studies instead demonstrated elevated pain tolerance while pain ratings remained unaffected (Hayes et al., 1999; Keogh et al., 2005) or even were reduced (Masedo and Esteve, 2007). Only one study (Braams et al., 2012) showed reduced pain ratings when investigating acceptance-based strategies by using brief pain stimuli instead of pain tolerance tasks. Future studies should incorporate both subjective pain processing and behavioral pain measures.

### Effects of the Acceptance-Based Strategy on Physiological Pain Responses

In our study, we recorded HR and SC as psychophysiological pain responses (Loggia et al., 2011). Contrary to our hypothesis, analysis of SC did not show a significant difference between the acceptance and control condition. However, we found general SC responses following the pain stimulation around 6 s after pain onset, similar to previous studies (Breimhorst et al., 2011). According to the meta-analysis by Kohl et al. (2012), findings regarding the influence of acceptance-based strategies on physiological correlates of emotion and pain are mixed. Several studies investigating acceptance-based strategies in the context of *emotion* regulation did not find any effects on HR (Eifert and Heffner, 2003; Dunn et al., 2009; Erisman and Roemer, 2010) or SC (Eifert and Heffner, 2003; Campbell-Sills et al., 2006; Erisman and Roemer, 2010). This indicates that accepting a negative affective state, which might also include pain, does not necessarily reduce physiological arousal (Kohl et al., 2012). Loggia et al. (2011) found that HR was a better predictor of pain ratings than SC, which might explain the different effects of the acceptance-based strategy on SC and HR in the present study.

We found the HR to be significantly lower in the acceptance compared to the control condition during cue offset, 3 s after the 10 s pain stimulation. This might indicate that acceptance-based strategies take some time to evolve their effect. Dan-Glauser and Gross (Dan-Glauser and Gross, 2015) did not find any differences between an acceptance-based and a control condition on negative emotions (8 s picture presentation) and concluded that acceptance-based strategies step in rather late in the emotion formation process (Gross, 1998). Similarly, our results might also reflect a later onset of acceptance-based strategy effects on pain. Alternatively, the more pronounced deceleration of the HR in the acceptance condition could reflect a faster recovery from pain. Temporal dynamics in subjective and physiological measures might become more evident in a longer tonic pain stimulation. Thus, different pain durations should be incorporated in future research. Furthermore, larger sample sizes might be helpful in investigating physiological responses, especially SC signals.

The questions remain, whether more training of acceptance-based strategies (Erisman and Roemer, 2010) and more detailed instructions (McMullen et al., 2008) might lead to even clearer subjective and physiological effects. Future research should systematically vary the amount of training prior to the experiment to detect critical aspects underlying the successful use of acceptance-based strategies.

### Limitations and Outlook

The present results showed that the use of acceptance-based pain regulation was associated with reductions of subjective and physiological pain responses. The effect of acceptance on psychophysiological pain measures might be further explored using different pain stimulation intensities and modalities or endogenous pain inhibitory indices (Horn-Hofmann et al., 2018). Furthermore, it might be worthwhile comparing an acceptance-based strategy with other well-established regulation strategies such as reappraisal or distraction to identify shared and unique processes involved in the regulation of pain. Given potential gender differences in pain processing and coping (Fillingim et al., 2009), it would be interesting to address them in future pain regulation studies providing sufficiently large sample sizes.

We carefully instructed participants to follow all experimental instructions, and their compliance is supported by both our results and manipulation check. Nevertheless, we cannot completely rule out the use of acceptance during the control condition or alternative coping strategies (Cioffi and Holloway, 1993). In future studies, more detailed post experimental surveys and additional measures of experimental adherence should be employed to detect potential confounds.

An expectancy toward a certain outcome plays a crucial role in the effectiveness of mindfulness and acceptance-based strategies (Brown and Jones, 2010; Zeidan et al., 2012), hence eliminating its effect would be difficult let alone meaningless. However, it would be interesting for future research to capture participants’ expectations regarding the effectiveness of pain regulation strategies systematically.

Although HR and SC serve as reliable psychophysiological indicators of pain responses (Rhudy et al., 2009; Loggia et al., 2011), they undoubtedly capture only a small portion of the processes involved in emotion and pain regulation (Kohl et al., 2012). HR variability, for instance, is a well-established measure of ER (Appelhans and Luecken, 2006) and might be a promising index for the regulation of pain unpleasantness (Appelhans and Luecken, 2008). However, analyzing HR variability would be at the expense of capturing temporal dynamics as its calculation requires prolonged intervals (Shaffer and Ginsberg, 2017).

In the present study, we did not continuously measure subjective pain to avoid distraction from the pain stimulation and to prevent disruption of strategy usage. Nevertheless, continuous ratings [e.g., with rating dials (Hutcherson et al., 2005)] in ER research reliably measured ongoing emotions without interfering with them or the strategy application (Hutcherson et al., 2005; Dan-Glauser and Gross, 2011). Incorporating continuous pain ratings might be a promising tool for future regulation research.

We did not find any associations between ER styles or other psychological factors such as anxiety or pain sensitivity and the effectiveness of the acceptance-based strategy in modulating pain. Yet, individual differences in preferred ER styles could still play a critical role in the effectiveness of pain regulation strategies. This might be especially relevant for research on chronic pain since the habitual use of maladaptive ER strategies, like experiential suppression, could represent a risk factor for pain chronification (Koechlin et al., 2018). Thus, studies using larger sample sizes are necessary to explore the role of psychological traits for pain regulation.

Future research should consider translating similar experimental designs – including carefully prepared control conditions – to chronic pain populations, providing a deeper understanding of the mechanisms involved in successful pain acceptance and advance the development of psychological interventions for chronic pain.

## Data Availability Statement

The datasets generated for this study are available on request to the corresponding author.

## Ethics Statement

The studies involving human participants were reviewed and approved by the institutional review board of the medical faculty of the University of Würzburg. The patients/participants provided their written informed consent to participate in this study.

## Author Contributions

VH organized the database, performed the statistical analysis, and wrote the first draft of the manuscript. All authors wrote sections of the manuscript, contributed to conception and design of the study, manuscript revision, and read and approved the submitted version.

## Conflict of Interest

The authors declare that the research was conducted in the absence of any commercial or financial relationships that could be construed as a potential conflict of interest.

## Abbreviations

ACT: Acceptance and Commitment Therapy
ECG: electrocardiograph
ER: emotion regulation
HR: heart rate
MCS: manipulation check survey
PT: pain threshold
SC: skin conductance
VAS: visual analog scale.
