# Supplementary material for: Acceptance-Based Emotion Regulation Reduces Subjective and Physiological Pain Responses
Source: Front Psychol. 2020 Jun 30;11:1514. doi: 10.3389/fpsyg.2020.01514 (PMC7338768; doi:10.3389/fpsyg.2020.01514)
Supplement: Supplementary file 1 [file Data_Sheet_1.PDF]

frontiers

Supplementary Material

Additional information regarding the conceptualization of acceptance-based strategies

In his process model of ER, Gross (1) differentiates between two kinds of ER strategies: the antecedent-focused and the response-focused strategies. The antecedent-focused strategies summarize all strategies that act before an emotional reaction is actually generated, whereas the response-focused strategies target the already ongoing emotional reaction and aim at changing its experiential, behavioral and physiological outcome. According to the process model, the earlier a regulation strategy is engaged during the emotion-generative process, the less cognitive resources are needed and the easier the regulatory goal is achieved (2, 3).

In the original version of the process model, acceptance-based strategies are not explicitly represented, however there is an ongoing debate whether acceptance-based regulatory strategies can be classified within the process model as an antecedent- or response-focused ER strategy (4-7). On one hand, acceptance is argued to be a response-focused strategy as it aims at engaging in the already generated, ongoing emotional reactions (4). On the other hand, acceptance also entails antecedent components, targeting the cognitive change of the emotion-eliciting event (5). Similarly, Wolgast and Lundh (7) concluded that acceptance has both antecedent- and response-focused components.

The commonly applied theoretical approach of acceptance-based strategies in ER research (5, 8, 9) and pain regulation research (10-12) refers to the Acceptance and Commitment Therapy (ACT) (13), which also served as the basis for our conceptualization of the acceptance-based strategy in the present study. We integrated different components addressing acceptance, defusion, and mindfulness as these three core ACT processes were applied repeatedly in several studies on emotion and pain regulation (5, 10, 11, 14) and we adapted the instructions accordingly. As outlined above, one might argue that our conceptualization of acceptance might include cognitive change and thus resemble to some degree reappraisal of the affective component of the pain stimulation. Nevertheless, our instruction clearly does not involve a reinterpretation of sensory characteristics or the significance of the pain stimulation itself, as usually performed in pain regulation research (15). Moreover, when referring to the idea of acceptance, one might be reminded of acceptance in the context of religion, however this not the case in the present study or current research on pain and emotion more generally, even though past conceptualizations of ACT had a stronger spiritual foundation (16).

References

1. Gross JJ. Antecedent- and response-focused emotion regulation: Divergent consequences for experience, expression, and physiology. *J Pers Soc Psychol* (1998) 74(1):224-37. doi: 10.1037/0022-3514.74.1.224. PubMed PMID: 1997-38342-016.

2. Gross JJ. Emotion regulation: Affective, cognitive, and social consequences. *Psychophysiology* (2002) 39(3):281-91. doi: 10.1017/S0048577201393198. PubMed PMID: WOS:000175419200002.

3. John OP, Gross JJ. Healthy and unhealthy emotion regulation: Personality processes, individual differences, and life span development. *Journal of Personality* (2004) 72(6):1301-33. Epub 2004/10/29. doi: 10.1111/j.1467-6494.2004.00298.x. PubMed PMID: 620556294.

4. Hofmann SG, Asmundson GJG. Acceptance and mindfulness-based therapy: New wave or old hat? *Clin Psychol Rev* (2008) 28(1):1-16. doi: 10.1016/j.cpr.2007.09.003. PubMed PMID: 2008-00305-003.

5. Hofmann SG, Heering S, Sawyer AT, Asnaani A. How to handle anxiety: The effects of reappraisal, acceptance, and suppression strategies on anxious arousal. *Behav Res Ther* (2009) 47(5):389-94. doi: 10.1016/j.brat.2009.02.010. PubMed PMID: 2009-06419-007.

6. Liverant GI, Brown TA, Barlow DH, Roemer L. Emotion regulation in unipolar depression: The effects of acceptance and suppression of subjective emotional experience on the intensity and duration of sadness and negative affect. *Behav Res Ther* (2008) 46(11):1201-9. doi: 10.1016/j.brat.2008.08.001. PubMed PMID: 2008-14867-003.

7. Wolgast M, Lundh L-G, Viborg G. Cognitive reappraisal and acceptance: An experimental comparison of two emotion regulation strategies. *Behav Res Ther* (2011) 49(12):858-66. Epub 2011/10/18. doi: 10.1016/j.brat.2011.09.011. PubMed PMID: 2011-25847-005.

8. Asnaani A, Sawyer AT, Aderka IM, Hofmann SG. Effect of suppression, reappraisal, and acceptance of emotional pictures on acoustic eye-blink startle magnitude. *Journal of Experimental Psychopathology* (2013) 4(2):182-93. doi: 10.5127/jep.028112. PubMed PMID: 2013-44049-006.

9. Dan-Glauser ES, Gross JJ. The temporal dynamics of emotional acceptance: Experience, expression, and physiology. *Biol Psychol* (2015) 108:1-12. doi: 10.1016/j.biopsycho.2015.03.005. PubMed PMID: WOS:000353996100001.

10. Braams BR, Blechert J, Boden MT, Gross JJ. The effects of acceptance and suppression on anticipation and receipt of painful stimulation. *J Behav Ther Exp Psychiatry* (2012) 43(4):1014-8. Epub 2012/05/15. doi: 10.1016/j.jbtep.2012.04.001. PubMed PMID: 22580070.

11. Kohl A, Rief W, Glombiewski JA. Acceptance, Cognitive Restructuring, and Distraction as Coping Strategies for Acute Pain. *J Pain* (2013) 14(3):305-15. doi: 10.1016/j.jpain.2012.12.005. PubMed PMID: WOS:000316038200010.

12. Masedo AI, Esteve MR. Effects of suppression, acceptance and spontaneous coping on pain tolerance, pain intensity and distress. *Behav Res Ther* (2007) 45(2):199-209. doi: 10.1016/j.brat.2006.02.006. PubMed PMID: 16569396.

13. Hayes SC, Luoma JB, Bond FW, Masuda A, Lillis J. Acceptance and commitment therapy: Model, processes and outcomes. *Behav Res Ther* (2006) 44(1):1-25. doi: 10.1016/j.brat.2005.06.006. PubMed PMID: 621022521.

14. Hayes SC, Bissett RT, Korn Z, Zettle RD, Rosenfarb IS, Cooper LD, et al. The impact of acceptance versus control rationales on pain tolerance. *Psychol Rec* (1999) 49(1):33-47. doi: 10.1007/BF03395305. PubMed PMID: WOS:000079288200003.

15. Lapate RC, Lee H, Salomons TV, van Reekum CM, Greischar LL, Davidson RJ. Amygdalar function reflects common individual differences in emotion and pain regulation success. *J Cogn Neurosci* (2012) 24(1):148-58. doi: 10.1162/jocn_a_00125. PubMed PMID: 21861676; PubMed Central PMCID: PMC3298185.

16. Hayes SC. Acceptance, mindfulness, and science. *Clinical Psychology: Science and Practice* (2002) 9(1):101-6. PubMed PMID: 619823971.
